# Placenta-Expanded Stromal Cell Therapy in a Rodent Model of Simulated Weightlessness

**DOI:** 10.3390/cells10040940

**Published:** 2021-04-19

**Authors:** Linda Rubinstein, Amber M. Paul, Charles Houseman, Metadel Abegaz, Steffy Tabares Ruiz, Nathan O’Neil, Gilad Kunis, Racheli Ofir, Jacob Cohen, April E. Ronca, Ruth K. Globus, Candice G. T. Tahimic

**Affiliations:** 1 Universities Space Research Association, Columbia, MD 21046, USA; linda.rubinstein@gmail.com (L.R.); amber.paul@erau.edu (A.M.P.); 2 Space Biosciences Division, NASA Ames Research Center, Moffett Field, CA 94035, USA; charles.j.houseman@nasa.gov (C.H.); metadel.f.abegaz@gmail.com (M.A.); stabaresruiz@alumni.scu.edu (S.T.R.); nnoneil22@gmail.com (N.O.); jacob.cohen-1@nasa.gov (J.C.); april.e.ronca-1@nasa.gov (A.E.R.); ruthglobus@yahoo.com (R.K.G.); 3 Department of Human Factors and Behavioral Neurobiology, Embry-Riddle Aeronautical University, Daytona Beach, FL 32114, USA; 4 Blue Marble Space Institute of Science, Seattle, WA 98154, USA; 5 Pluristem Ltd., Haifa 31905, Israel; giladk@pluristem.com (G.K.); racheli@pluristem.com (R.O.); 6 Wake Forest Medical School, Winston-Salem, NC 27101, USA; 7 KBR, Houston, TX 77002, USA; 8 Department of Biology, University of North Florida, Jacksonville, FL 32224, USA

**Keywords:** spaceflight, hindlimb unloading, PLX-PAD cells, placenta, cell therapy, immune system, bone loss, muscle atrophy, cytokines, hippocampus, CNS

## Abstract

Long duration spaceflight poses potential health risks to astronauts during flight and re-adaptation after return to Earth. There is an emerging need for NASA to provide successful and reliable therapeutics for long duration missions when capability for medical intervention will be limited. Clinically relevant, human placenta-derived therapeutic stromal cells (PLX-PAD) are a promising therapeutic alternative. We found that treatment of adult female mice with PLX-PAD near the onset of simulated weightlessness by hindlimb unloading (HU, 30 d) was well-tolerated and partially mitigated decrements caused by HU. Specifically, PLX-PAD treatment rescued HU-induced thymic atrophy, and mitigated HU-induced changes in percentages of circulating neutrophils, but did not rescue changes in the percentages of lymphocytes, monocytes, natural killer (NK) cells, T-cells and splenic atrophy. Further, PLX-PAD partially mitigated HU effects on the expression of select cytokines in the hippocampus. In contrast, PLX-PAD failed to protect bone and muscle from HU-induced effects, suggesting that the mechanisms which regulate the structure of these mechanosensitive tissues in response to disuse are discrete from those that regulate the immune- and central nervous system (CNS). These findings support the therapeutic potential of placenta-derived stromal cells for select physiological deficits during simulated spaceflight. Multiple countermeasures are likely needed for comprehensive protection from the deleterious effects of prolonged spaceflight.

## 1. Introduction

Spaceflight causes rapid changes in organs and tissues that may pose health risks to mission crew during long duration space travel. These risks arise from the physical environment of spaceflight, which includes a combination of unique factors such as microgravity, ionizing radiation, and social isolation, amongst others. Microgravity during spaceflight causes a cephalad fluid shift and musculoskeletal disuse, which can lead to bone loss, muscle atrophy and cardiovascular and neurovestibular changes [1,2,3,4,5,6,7,8,9]. In addition, spaceflight results in immune dysregulation and viral reactivation [10,11]. Crewmembers on a six-month mission to the International Space Station (ISS) display elevated levels of inflammatory cytokines and impaired T/NK cell function [12], while shorter duration missions (9–15 days) result in humoral and monocytic immune impairments, and low-grade inflammation [13,14]. We previously showed that long duration HU combined with isolation (30 days) reduces spleen mass and the percentages of circulating CD4+ T cells and neutrophils [3]. HU also elevates the neutrophil to lymphocyte ratio (NLR), as does spaceflight [15], indicative of increased inflammation.

Placental stromal cells have demonstrated therapeutic efficacy in various tissue injury models [16,17,18,19,20]. In this study, we sought to determine whether human PLacenta eXpanded mesenchymal-like stromal cells (PLX-PAD) can mitigate the adverse effects of simulated microgravity in several physiological systems known to be adversely affected by spaceflight, including the musculoskeletal, immune, and central nervous systems (CNS). PLX-PAD cells are derived from the maternal component of the full-term placenta. PLX-PAD cells secrete cytokines and other factors that modulate the immune system, promote tissue regeneration, angiogenesis, hematopoiesis, and neurogenesis [20,21,22,23]. In addition, they do not trigger major host immune responses, enabling allogeneic use and direct testing in animals that have intact immune systems [24]. PLX-PAD also promotes the growth of collateral blood vessels in damaged tissue, tissue regeneration, and immune system modulation in response to injury by the secretion of pro-angiogenic factors, such as angiogenin and angiopoietin-1 [20]. In a rodent model of critical limb ischemia, the administration of PLX-PAD was shown to improve blood flow, capillary density, and limb function, as well as reducing oxidative stress [20]. PLX-PAD also improves recovery following tendon injuries, possibly through the formation of new collagen [25]. In a clinical setting, PLX-PAD induced significant recovery of muscle strength and volume in a phase II clinical trial in patients undergoing total hip replacement [26]. We selected PLX-PAD as the principal cell line for testing therapeutic efficacy based on its well-established ability to improve healing of mesenchymal tissues. Here, we show that PLX-PAD cells prevent HU-induced thymic atrophy, as well as changes in the percentage of select circulating immune cell populations. Further, PLX-PAD cells partially prevent the effect of HU on the expression of select hippocampal cytokines. However, PLX-PAD cells did not protect from HU-induced musculoskeletal deficits.

## 2. Materials and Methods

**Figure 1:**
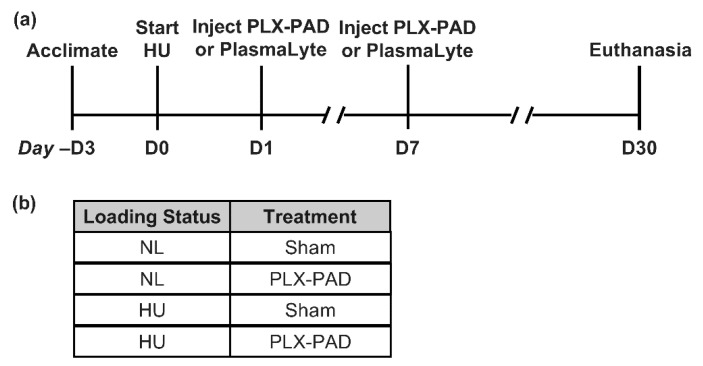
Study design and timing of procedures. (**a**) Timeline of treatments and procedures in this study. Hindlimb unloading (HU) and normally loaded (NL, control) groups received two intramuscular injections of PLX-PAD or PlasmaLyte (Sham) and euthanized 30 days after onset of HU. (**b**) Summary of experimental groups.

Animals and experimental design. All animal studies were conducted with prior approval from the NASA Ames Institutional Animal Care and Use Committee (IACUC). Three days prior to HU, 16-week-old female *C57BL/6NJ* mice (Jackson Laboratories) were single-housed in the HU caging system [8,27]. Normally loaded (NL) controls were housed in vivarium cages and were handled the same as HU mice, except for application of traction tape and tail suspension. Room temperature was maintained at 25–27 °C and animals were supplied fresh nestlets (Ancare, Cat# NES3600) daily, as enrichment. HU for 30 days was conducted as described previously [3]. One day after the onset of HU, all mice were injected with 50 μL of PLX-PAD (1 × 10^6^ cells) or PlasmaLyte 148 (Baxter) in each quadricep. A second injection (as above) was given on day 7 of HU. PlasmaLyte is referred to as a Sham treatment (the carrier for PLX-PAD, containing 5% human serum albumin and 10% dimethylsulfoxide (DMSO)). Animals were euthanized 30 days after the onset of HU via CO_2_ inhalation and cervical dislocation followed by blood collection from the vena cava and removal of tissues. All tissues were weighed shortly thereafter. Peripheral blood was collected in K3 EDTA tubes (Sarstedt, Cat# 41.1395.105). Centrifugation was performed at 1000× *g* at room temperature for 10 min. Plasma was collected and immediately flash frozen. Cellular components were used for flow cytometry analyses. Refer to Figure 1 for groups and experimental timeline. Sample sizes are *n* = 12 per group unless otherwise noted.

PLX cell preparation. The details of the PLX production process and phenotypic characterization of the cells have been described previously [28,29]. In brief, placentae were collected from healthy women undergoing an elective caesarean section and processed at Pluristem Ltd., Haifa (Israel). Adherent cells were first selected and grown in two-dimensional culture flasks for approximately one month and later expanded in a dedicated bioreactor system on a fibrous carrier material for one week. Cells were shipped frozen, thawed immediately prior to the experiment, and injected in vivo without further cultivation steps as described previously [24].

Flow cytometry. Preparation and flow cytometric analysis of peripheral WBCs were performed as described previously [3]. Antibodies utilized for analysis were as follows: anti-CD45 (11-0451-82), anti-Ly6g (12-9668-82), anti-CD11b (25-0112-82), anti-CD3 (11-0031-85), anti-CD4 (12-0041-85), anti-CD8 (MCD0831), anti-NK1.1 (17-5941-82), and anti-CD69 (48-0691-82). Unstained and single-stained compensation controls were used, and all antibodies were purchased from Thermo Fisher Scientific. Data was acquired using a BD FACSMelody and FlowJo software (version 10.3.0) was used for cytometric analysis, both from BD Biosciences.

Cytokine analysis. The left hemisphere of the brain was microdissected immediately after euthanasia. The hippocampus was flash frozen and stored at −80 °C. Hippocampal tissue was homogenized in cold mild lysis buffer (Tris 50 mM, NaCl 150 mM, Igepal 1%, Protease Inhibitors all from Millipore-Sigma). Centrifugation was performed at 1000× *g* for 10 min at 4 °C. Supernatants were collected, aliquots created and then frozen at −80 °C until analysis. Cytokine protein levels in hippocampal homogenates were measured using a 44-Plex MD44 Mouse Cytokine Array/Chemokine Array (Eve Technologies). Concentration standards were included for each cytokine. Hippocampal cytokine levels were normalized to total protein content as measured by BCA assay (Thermo Fisher). Undiluted plasma was analyzed for cytokine protein abundance using the same cytokine array.

Corticosterone analysis. Plasma was diluted to 1:100 and analyzed using a corticosterone ELISA kit (Abcam, ab108821) per manufacturer’s recommendations.

Microcomputed tomography (μCT). The Skyscan 1272 system and associated software (Bruker) were used to generate images and analyze cortical and cancellous compartments of the tibia. To assess cancellous bone microarchitecture, the proximal tibia was scanned at a resolution of 3.5 μm. A 399 μm region of interest (ROI) was used, located 350 μm distal to the growth plate. Cancellous parameters analyzed included percent bone volume (BV/TV, %), trabecular thickness (Tb.Th, mm), trabecular spacing (Tb.Sp, mm), trabecular number (Tb.N., 1/mm), and connectivity density (Conn.Dn.). A 300 μm ROI located 2304 μm proximal to the tibio-fibular junction was used to analyze cortical bone microarchitecture. Parameters examined included cortical thickness (Ct.Th, mm), cortical area (Ct.Ar., mm^2^), and cortical volume (Ct.Vol., mm^3^), periosteal perimeter (Ps.Pm., mm), endosteal perimeter (Es.Pm, mm), polar moment of inertia (pMOI, mm^4^), and tissue mineral density (TMD, g·cm^3^).

Statistical analysis. Statistical analyses were performed using JMP software version 13.1.0 (SAS Institute Inc., Cary, NC, USA). Tests for equal variance and normality were conducted using Levene’s test and Shapiro-Wilk goodness of fit test, respectively. If the variances were equal and normality confirmed, a one-way analysis of variance (ANOVA) was performed across NL Sham, HU Sham, NL PLX-PAD and HU PLX-PAD groups. If unequal variance or non-normal distribution was observed, a nonparametric Wilcoxon all pairs test was conducted. Unless otherwise stated, data shown are mean +/− standard deviation. GraphPad Prism software (version 9.0.1) was used to generate figures.

## 3. Results

**Figure 2:**
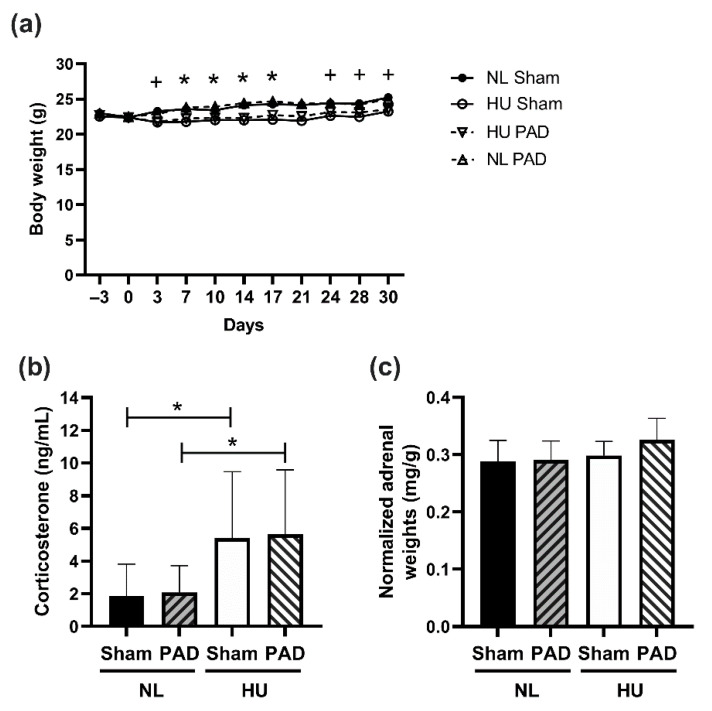
Body weights of experimental groups and measures of neuroendocrine stress. (**a**) Body weights from acclimation (Day −3) to Day 30 of hindlimb unloaded (HU) mice and normally loaded (NL) controls. Repeated measures ANOVA was performed. + Statistically significant difference between NL Sham vs. HU Sham at *p* < 0.05; * Statistically significant difference between NL Sham vs. HU Sham and NL PAD vs. HU PAD at *p* < 0.05. Sample sizes are: NL Sham (*n* = 12), NL PAD (*n* = 12), HU Sham (*n* = 11), and HU PAD (*n* = 9). Statistically significant at *p* < 0.05 by one-way ANOVA with Tukey post-hoc test. (**b**) Plasma corticosterone levels at Day 30 post-HU. NL Sham (*n* = 11), NL PAD (*n* = 12), HU Sham (*n* = 11), and HU PAD (*n* = 10). A nonparametric Wilcoxon all pairs test was performed; statistically significant at *p* < 0.05. (**c**) Total weights of left and right adrenals normalized to body weight. ‘PAD’ denotes PLX-PAD administration. Sample sizes are: NL Sham (*n* = 12), NL PAD (*n* = 12), HU Sham (*n* = 9), and HU PAD (*n* = 9). Statistically significant at *p* < 0.05 by one-way ANOVA with Tukey post-hoc test.

We first assessed the effects of PLX-PAD cell administration on select measures of stress response. Consistent with our previous observations [3], the HU Sham group had ~6% decrement in body weight compared to NL Sham controls from day 3 of unloading (except for day 21) (Figure 2a). PLX-PAD had a mild effect in mitigating body weight decrements due to HU on days 3, 24, 28 and 30 (Figure 2a). HU increased circulating corticosterone levels in the Sham group compared to NL Sham controls, and HU caused similar increases in PLX-PAD treated mice, compared to their corresponding controls (Figure 2b). Adrenal weights normalized to body weights were comparable across all experimental groups (Figure 2c). Collectively, these results indicate that treatment with PLX-PAD did not exacerbate the effects of HU on these indices of stress.

**Figure 3:**
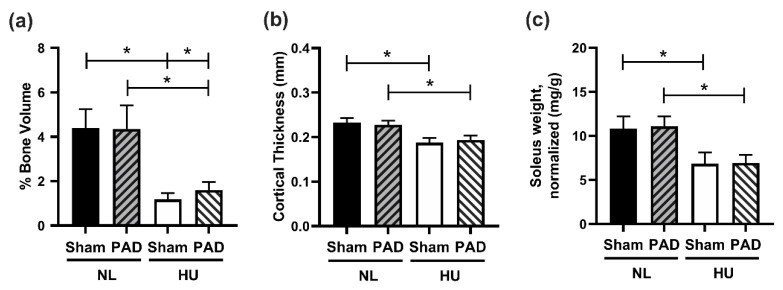
Musculoskeletal measurements. (**a**,**b**) μCT analysis of mouse tibiae. (**a**) Percent cancellous bone volume (BV/TV, %). NL Sham (*n* = 12), NL PAD (*n* = 12), HU Sham (*n* = 10), and HU PAD (*n* = 10). * Statistically significant at *p* < 0.05 by nonparametric Wilcoxon all pairs test at *p* < 0.05. (**b**) Cortical thickness (Ct.Th.). NL Sham (*n* = 11), NL PAD (*n* = 12), HU Sham (*n* = 11), and HU PAD (*n* = 10). * Statistically significant at *p* < 0.05 by one-way ANOVA with Tukey post-hoc test. (**c**) Right soleus weight normalized to body weight. NL Sham (*n* = 12), NL PAD (*n* = 12), HU Sham (*n* = 11), and HU PAD (*n* = 9). * Statistically significant at *p* < 0.05 by one-way ANOVA with Tukey post-hoc test.

We next assessed whether PLX-PAD can protect from HU-induced musculoskeletal deficits. HU caused the anticipated deficits in cancellous and cortical bone parameters, as measured by microcomputed tomography (μCT). It also reduced cancellous bone parameters including bone volume (Figure 3a), trabecular thickness, trabecular number, connectivity density, and structural model index (SMI) (Supplementary Figure S1a–d), and increased trabecular spacing (Supplementary Figure S1e), with no changes in degree of anisotropy (DA) (Supplementary Figure S1f). In cortical tissue, HU decreased cortical thickness (Figure 3b), polar moment of inertia (MOI), and tissue mineral density (TMD) (Supplementary Figure S2a,b respectively). HU also increased endosteal perimeter and periosteal perimeter (Supplementary Figure S2c,d respectively). Administration of PLX-PAD did not prevent HU-induced changes in both cortical and cancellous bone structural parameters. Additionally, HU led to the expected decrement in soleus weight (normalized to body weight) in the sham groups, while PLX-PAD did not mitigate this effect (Figure 3c).

We also sought to determine the effects of PLX-PAD on osteogenic bone marrow stromal cells by measuring colony counts and mineralization in ex vivo cell culture (osteoblastogenesis) (Supplementary Figure S3, Supplementary Methods). We found that colony counts during the growth phase (days 5 and 9, Supplementary Figure S3a,b) and percent mineralization at the fully differentiated phase (day 22, Supplementary Figure S3c) were comparable across all four experimental groups.

**Figure 4:**
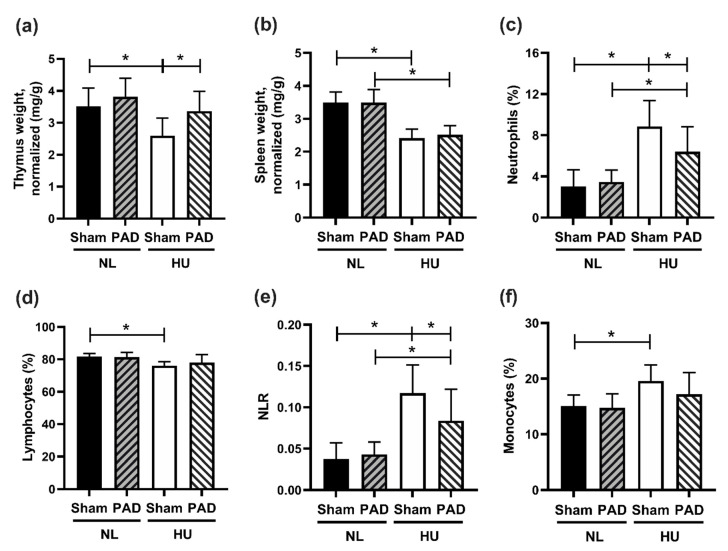
Immune organ weight and cell profiling at day 30 post-HU. (**a**) Thymus and (**b**) spleen weights normalized to body weights at day 30 post-HU. (**c**–**f**) Results from flow cytometry of whole blood collected from mice at day 30 post-HU. (**c**) Neutrophil within leukocytes population percentages (%, Ly6g+CD11b+/CD45+), (**d**) Lymphocytes within leukocyte population percentage (%, CD11b−/CD45+), (**e**) Neutrophil to lymphocyte ratio (NLR), and (**f**) Total monocyte to leukocyte population percentage (%, CD11b+/CD45+). NL Sham (*n* = 12), NL PAD (*n* = 12), HU Sham (*n* = 11), and HU PAD (*n* = 9). * Statistically significant at *p* < 0.05 by one-way ANOVA with Tukey post-hoc test.

We next determined the effects of PLX-PAD on select immune responses to HU. HU Sham animals had reduced thymus weights (normalized to body weight) compared to NL Sham controls, while there were no statistically significant differences between HU PLX-PAD and NL PLX-PAD animals (Figure 4a). HU led to a reduction in spleen weights (normalized to body weights) compared to NL controls in both Sham and PLX-PAD treated animals (Figure 4b). Neutrophils were elevated in HU Sham versus NL Sham groups, as well as HU PLX-PAD versus NL PLX-PAD groups. However, HU PLX-PAD mice had reduced neutrophils compared to the HU Sham group (Figure 4c). Percent lymphocytes in HU Sham animals was also decreased modestly compared to Sham NL controls, whereas PLX-PAD-treated mice had comparable percentages (Figure 4d). An elevated neutrophil to lymphocyte ratio (NLR) is associated with subclinical inflammation [30] during both spaceflight and HU [15,31]. We found that HU of sham animals led to a higher NLR compared to NL Sham controls (Figure 4e), as we previously described [15,31]. PLX-PAD conferred a 30% protective effect against changes in NLR (Figure 4e). Monocyte and NK/NKT cell percentages were elevated in HU Sham animals compared to NL Sham controls (Figure 4f and Supplementary Figure S4a, respectively). However, HU mice treated with PLX-PAD had comparable percentages of monocytes and NK/NKT cells to the Sham HU mice, suggesting no effect of PLX-PAD treatment on these cell populations (Figure 4f and Supplementary Figure S4a, respectively). HU Sham animals had decreased % T helper cells versus NL Sham, and PLX-PAD administration had no effect (Supplementary Figure S4b). Percentages of T cytotoxic and activated T cells were comparable across the four groups (Supplementary Figure S4c,d).

Since cytokines regulate the immune system, a 44-cytokine protein panel was used to analyze cytokine expression levels in plasma following 30 days of HU (Supplementary Tables S1 and S2). HU downregulated two cytokines, i.e., TIMP-1 and IL-6, compared to NL controls. PLX-PAD prevented HU effects only in IL-6 and not in TIMP-1. IL-5 protein levels were unchanged by HU in the Sham group; however, PLX-PAD treatment in NL mice led to increased IL-5 levels compared to the corresponding Sham-treated group. In addition, PLX-PAD treatment in HU mice reduced IL-5 levels relative to NL PLX-PAD controls (Supplementary Table S1).

**Figure 5:**
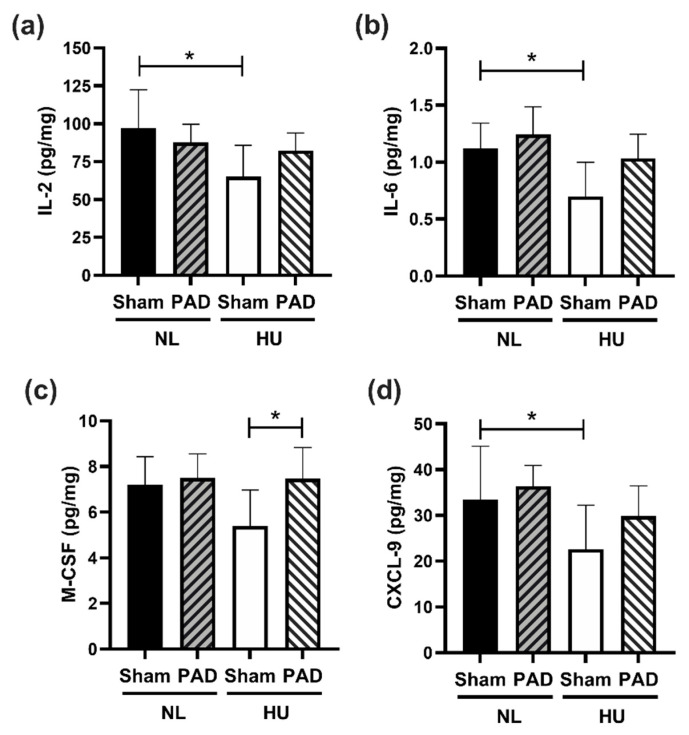
Protein levels of representative cytokines in hippocampus normalized to total protein content at day 30 post-HU. (**a**) IL-2, (**b**) IL-6 and (**c**) M-CSF and (**d**) CXCL-9. NL Sham (*n* = 8), NL PAD (*n* = 6), HU Sham (*n* = 8), and HU PAD (*n* = 8). * Statistically significant by one-way ANOVA and Tukey post-hoc test at *p* < 0.05.

To determine effects of PLX-PAD treatment in the CNS, we performed the same 44-cytokine protein expression panel on the hippocampus. Similar to our findings in plasma, there was an overall trend for lower cytokine levels in HU Sham compared to both NL groups and to HU PLX-PAD treated mice (Supplementary Tables S1 and S2). Compared to NL Sham controls, HU Sham animals showed downregulation of IL-2, IL-6, M-CSF, CXCL9 (Figure 5a–d respectively), IL-7, IL-13, IL-5, MCP-1 and EPO (Supplementary Tables S1 and S2). These HU effects were mitigated by PLX treatment in all these cytokines except for EPO. Examples of this mitigation include IL-2, IL-6, M-CSF and CXCL-9 (Figure 5a–d).

## 4. Discussion

The purpose of this study was to assess the efficacy of human placenta derived stromal cells, PLX-PAD, in mitigating various tissue deficits caused by simulated microgravity. We found that treatment with PLX-PAD was well tolerated both in NL and HU mice, as indicated by body weight measurements, corticosterone values, and immune cell profiling. HU in sham-treated mice led to expected musculoskeletal deficits, shifts in select immune cell populations, and changes in levels of cytokines in the plasma and hippocampus, while PLX-PAD reversed HU-induced changes in select immune and hippocampus measurements without mitigating musculoskeletal system deficits.

HU causes substantial decrements in bone structure and muscle mass [3,6], similar to findings from spaceflight [32,33]. For instance, the HU-induced decrease in soleus muscle weight observed in this study is consistent with previous reports from spaceflight and HU experiments [27,34,35], although muscle fiber typing was not performed in our study. Despite the regenerative effects of PLX-PAD in humans following injury [26], decrements in muscle and bone mass were not affected by PLX-PAD treatment, although there was a marginal improvement of bone volume per total volume in HU mice that received PLX-PAD. Treatment with PLX-PAD did not protect from HU-induced soleus muscle loss. In our hands, prolonged HU did not result in any impairments in colony formation and mineralization as evaluated using osteoblastogenesis assays. However, others report that long duration HU can cause a persistent decrement in these measures of osteoblastogenesis [36,37,38]. Differences in the results obtained in our study and theirs may be attributable to differences in species, sex, strain animal age, and/or osteogenic cell culture conditions.

Simulated weightlessness by HU caused atrophy of the spleen and thymus in sham-treated animals, consistent with other reports [3,39,40]. As mammals age, their thymus naturally atrophies; however, 30 days of HU caused enhanced atrophy, possibly in part due to activation of the hypothalamic-pituitary-adrenal (HPA) axis and concomitant elevated corticosterone [41,42]. In addition, it is also possible that neutrophil populations may accumulate in the spleen, which may concomitantly reduce T and B cell populations, as described in aging mice [43]. Although the mechanisms of thymic or splenic atrophy due to HU were not fully investigated in this study, reduced diversity of the peripheral T cell repertoire and number are possible, which could subsequently impact splenic weight and immune responses. Furthermore, it is possible that neutrophils associated with the spleen, thymus, and blood may have differing effector functions, since the location of immune cells directs immune function [44,45]. Therefore, phenotyping primary and secondary immune organs are needed to gain a better understanding of the immune profile of HU and corresponding PLX-PAD treated mice. Importantly, PLX-PAD treatment rescued thymic atrophy in HU mice, suggesting that PLX-PAD treatment may either delay or repair T-cell deficits, thereby providing immune support. Effective adaptive immunity is particularly important during extended duration spaceflight. Thus, more studies are needed to comprehensively evaluate the effects of PLX cells on adaptive immune responses in spaceflight models.

Immune cell phenotyping revealed that HU Sham animals had elevated neutrophils, monocytes, and NK cells; decreased T-helper cell and lymphocyte populations; and no change in T-cytotoxic and activated T-cells at 30 days post-HU compared to NL Sham controls. Results from immune cell profiling were generally consistent with our previous findings from a 30-day HU study involving similarly aged animals [3,15]. Interestingly, the administration of PLX-PAD partially mitigated some HU-induced changes in immune cell populations. Neutrophils were significantly reduced compared to HU Sham mice, suggesting that inflammation may have been suppressed in PLX-PAD treated HU mice. Elevated neutrophils in blood circulation may result from increased expression of chemokines related to neutrophil recruitment or a deficit in neutrophil apoptosis during HU. Nonetheless, neutrophilia or elevated neutrophils in blood are a marker for inflammatory disease development [46].

Interestingly however, when we survey the cytokine profile in the plasma, neutrophil-specific chemokine and pro-inflammatory cytokine were not observed to be elevated at 30 days post-HU, which may be due to the late timepoint of sample collection. Plasma chemokine/cytokines may have been significantly induced earlier during HU. Plasma cytokine findings were consistent with a previous report, where no changes due to HU were observed in protein levels of circulating GM-CSF, IFN-γ, IL-1α, IL-2, IL-4, IL-5, IL-6, IL-10, IL-17 and TNF-α, although the duration of HU was shorter (three weeks) and the experiment was performed on males that were skeletally immature (two months of age) [47].

DMSO was shown in prior PLX studies to have no effect (data not shown), while in other reports (not PLX-PAD), it was shown to have an immunomodulatory effect when administered to mice. Several studies have shown that DMSO administration decreases the populations of CD4+, CD8+, and IFN-γ-producing CD4+ and CD8+ T cells in the spleen [48], and reduces cytokine production [49]. Moreover, others have shown that DMSO treatment can decrease thymus weight by inducing apoptosis of thymocytes [50,51]. Based on the aforementioned studies and our observed results, the elevation in thymus weight and cytokine production in the HU model following PLX treatment is not likely to be attributed to DMSO administration.

The neutrophil to lymphocyte ratio (NLR) was recently characterized by our group as a marker for inflammation due to HU [15,31] and is also used as a marker for subclinical inflammation in a number of inflammatory diseases [52]. In our study, NLR was elevated in HU Sham mice; this effect was partially rescued by PLX-PAD treatment, suggesting therapeutic stromal cells may suppress inflammation during simulated weightlessness. Interestingly, a significant upregulation of IL-5 was displayed in PLX-PAD NL mice compared to Sham NL mice, suggesting that PLX-PAD treatment may enhance B-cell division, plasma cell formation, IgG secretion, and eosinophil activation [53].

For some of the cytokines tested, PLX-PAD treatment of the NL group induced an up regulation of cytokine levels. Injection of xenogenic human cells such as PLX cells to mice can induce an immune response that could explain such results. However, our previous data show that serum cytokines are only transiently elevated in some of the PLX-injected mice, and are reduced to normal levels after several days. Moreover, PLX-PAD administration in mice was shown to reduce cytokine levels and to decrease pathogenesis under inflammatory conditions, such as LPS-induced acute respiratory distress syndrome (ARDS model), Experimental Autoimmune Encephalomyelitis (EAE), and Graft vs. Host Disease (GvHD) model (data not shown). Hence, it is unlikely that a xenogenic response explains the obtained results.

Immune cells are important for the maintenance of the CNS, and play a central role in neurogenesis and in hippocampal-dependent spatial learning and memory [54]. Cytokines are produced by glial cells, T cells, and macrophages [55,56], and within meningeal layers and the choroid plexus of the brain. These cells interact within the CNS to determine the outcome of the inflammatory reaction [57,58]. It would be worthwhile to assess the effects of the experimental treatments in cytokine production in each of these cells. This, in turn, may provide insight into whether specific immune cell types can be targeted for countermeasure development.

Dysregulation of cytokine expression levels are associated with various CNS pathologies [59,60] and can alter the blood-brain barrier [61,62]. Indeed, altered cytokine signaling in the CNS is linked to neuroinflammation and cognitive changes. Cognitive impairment is a major risk for astronaut performance on long duration missions. Therefore, we sought to determine the cytokine profile in the hippocampus, as it is responsible for learning and memory [63]. Indeed, we found that HU downregulated nine out of 44 cytokines, including IL-2, IL-6, and CXCL-9; eight of these cytokine changes were mitigated by PLX-PAD treatment. IL-2 deficiency is associated with impaired learning in mice, similar to Alzheimer’s disease [64], while knockout of IL-2 leads to impaired spatial learning and memory [65] and morphological changes in the hippocampus [66]. This cytokine may therefore play a role in the cognitive impairments observed due to HU [67]. Conversely, elevated IL-6 in the CNS results in neuro-impairment through reduced synaptic plasticity in the hippocampus [68], while ablation of IL-6 in glial cells reduces exploratory behavior and decreases anxiety [69]. Although IL-6 is known to affect neurobehavior, the role of reduced IL-6 during HU in our study is unclear.

HU also reduced CXCL-9, a chemokine involved in monocyte and lymphocyte recruitment, suggesting possible impairment of immune cell infiltration and phagocytosis into the brain parenchyma [70]. Importantly, PLX-PAD treatment of HU mice reduced M-CSF levels compared to PLX-PAD NL controls, although Sham HU animals did not display significant differences compared to Sham NL. This may indicate that PLX-PAD increased the recruitment of monocytes which are involved in enhanced phagocytosis and clearance of dead or damaged cells in the brain.

While downregulation of traditional inflammatory markers in the brain parenchyma at 30 days post-HU was noted, this was not unexpected, as the brain may compensate or produce internal protection mediators that could dampen inflammation at this timepoint of collection. In the literature, there are multiple examples of cytokine downregulation which correlate with disruption of the immune system [71,72,73,74]. Most of these are chronic inflammatory conditions such as chronic pain or chronic fatigue syndrome. We consider 30 days of HU as a chronic experimental treatment, since a 30-day period corresponds to about 4.1% of the lifetime of a mouse (~3 years of an average human lifespan). While future experiments are needed, we posit that the cytokine responses observed likely reflect a chronic response, consistent with the aforementioned studies. Further, cytokines can display pleiotropic responses, and different organs can produce vastly different immune responses [44,45]. Other studies have reported reductions in cytokine levels at a later timepoint after injury, likely corresponding to the regenerative/repair process in tissues [17]. This possibility could be explored in future HU studies. More studies are required at earlier timepoints to identify the mechanisms involved. In addition, female mice were selected for this study. Future investigations are needed to determine the sex-dependence of experimental outcomes.

The finding that PLX-PAD failed to effectively protect bone structure and muscle mass from HU suggests that the signaling mechanisms underlying musculoskeletal deficits are discrete from those of the CNS and immune system. We reason that the molecular pathways of mechanosensing and signaling in bone and muscle evolved to enable physiological responses to mechanical loads in order to meet changing needs of the mobile organism. In contrast, the CNS and immune systems evolved robust and rapid mechanisms for healing and regeneration in response to injury. The different mechanisms and kinetics of these two systems probably play an important role in their different responses to PLX-PAD treatment.

## 5. Conclusions

In conclusion, we found that PLX-PAD cells partially protected mice from a subset of HU-induced immune and CNS changes, but did not effectively protect the musculoskeletal system. To our knowledge, this is the first report on the therapeutic potential of stromal/stem cells against HU-induced deficits. Our finding of partial mitigation in the HU model warrants further study, along with optimized dose/frequency of administration, and further assessment of strain- and sex-dependent responses to HU. We speculate that after additional testing, PLX-PAD treatment may provide a suitable therapeutic for minimizing injury or inflammation-driven tissue damage during long duration space travel.

## Supplementary Materials

The following are available online at https://www.mdpi.com/article/10.3390/cells10040940/s1, Table S1: Protein levels of cytokines in hippocampus and plasma per treatment group, Table S2: Summary of statistical analysis of hippocampal and plasma cytokines, Figure S1: Microcomputed tomography analysis of cancellous bone of tibia at 30 days post-HU, Figure S2: Microcomputed tomography analysis of cortical bone of tibiae at 30 days post-HU, Figure S3: Ex vivo osteoblastogenesis assay from bone marrow stromal cells (BMSCs) of HU and NL mice, Figure S4: Results from flow cytometry of whole blood collected from mice at day 30 post-HU, Supplementary Methods.
