# Supplementary material for: Placenta-Expanded Stromal Cell Therapy in a Rodent Model of Simulated Weightlessness
Source: Cells. 2021 Apr 19;10(4):940. doi: 10.3390/cells10040940 (PMC8073415; doi:10.3390/cells10040940)
Supplement: Supplementary file 1 [file cells-10-00940-s001.zip › Suppl Fig 1 revised.pdf]

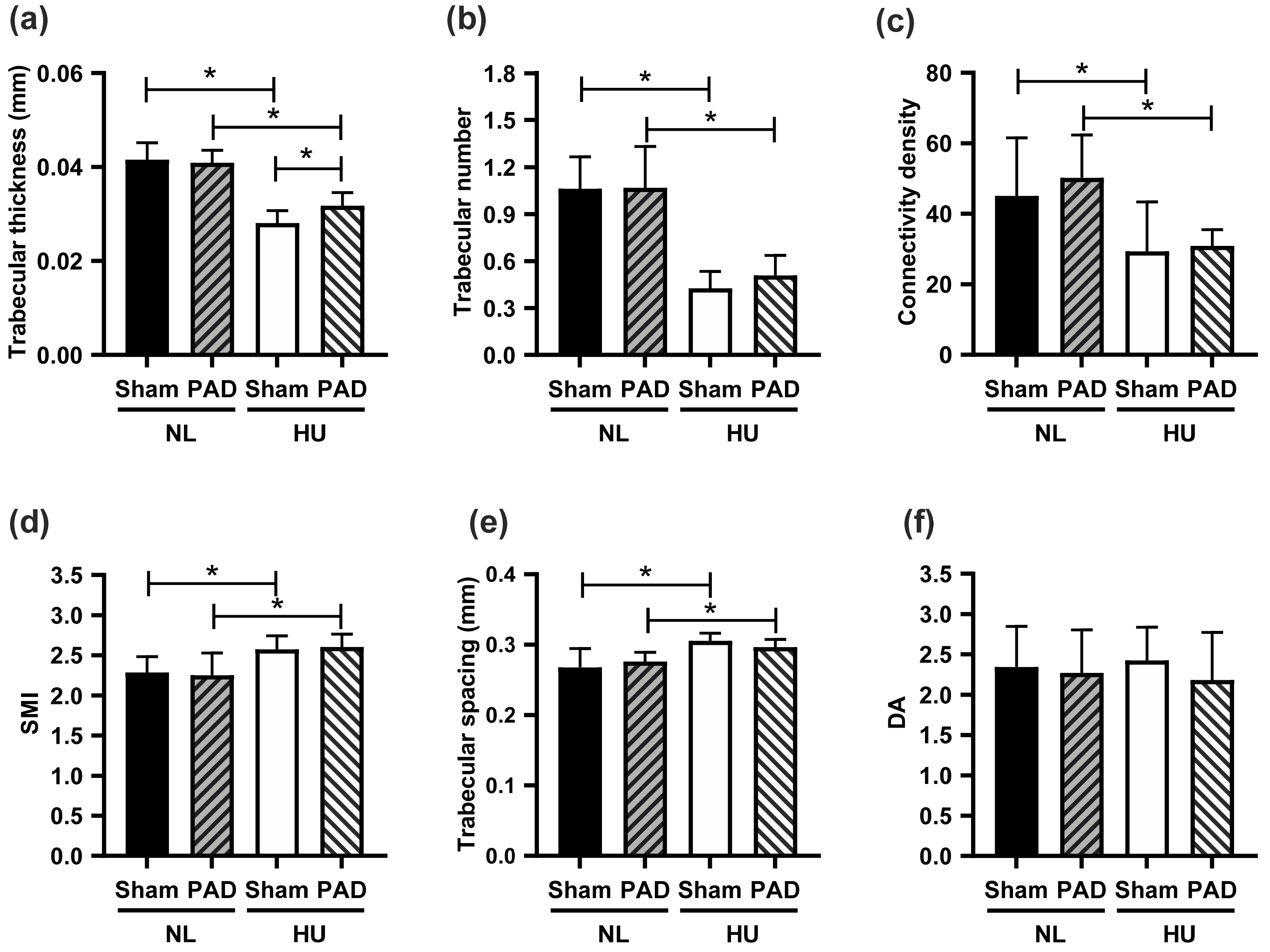

Supplementary Figure 1. Microcomputed tomography analysis of cancellous bone of tibia at 30 days post-HU. (a)Trabecular Thickness, Tb.Th.; (b) Trabecular Number, Tb.N.; (c) Connectivity Density, Conn.D.; (d) Structural Model Index, SMI; (e) Trabecular Spacing, Tb.Sp.; and (f) Degree of Anisotropy (DA). NL Sham (n=12), NL PAD (n=12), HU Sham (n=10), and HU PAD (n=10). (a, e, and f): *Statistically significant by one-way ANOVA and Tukey post-hoc test at p<0.05. (b-d): *Statistically significant by nonparametric Wilcoxon all pairs test at p<0.05.
