# Supplementary material for: Placenta-Expanded Stromal Cell Therapy in a Rodent Model of Simulated Weightlessness
Source: Cells. 2021 Apr 19;10(4):940. doi: 10.3390/cells10040940 (PMC8073415; doi:10.3390/cells10040940)
Supplement: Supplementary file 1 [file cells-10-00940-s001.zip › Suppl Fig 2 revised.pdf]

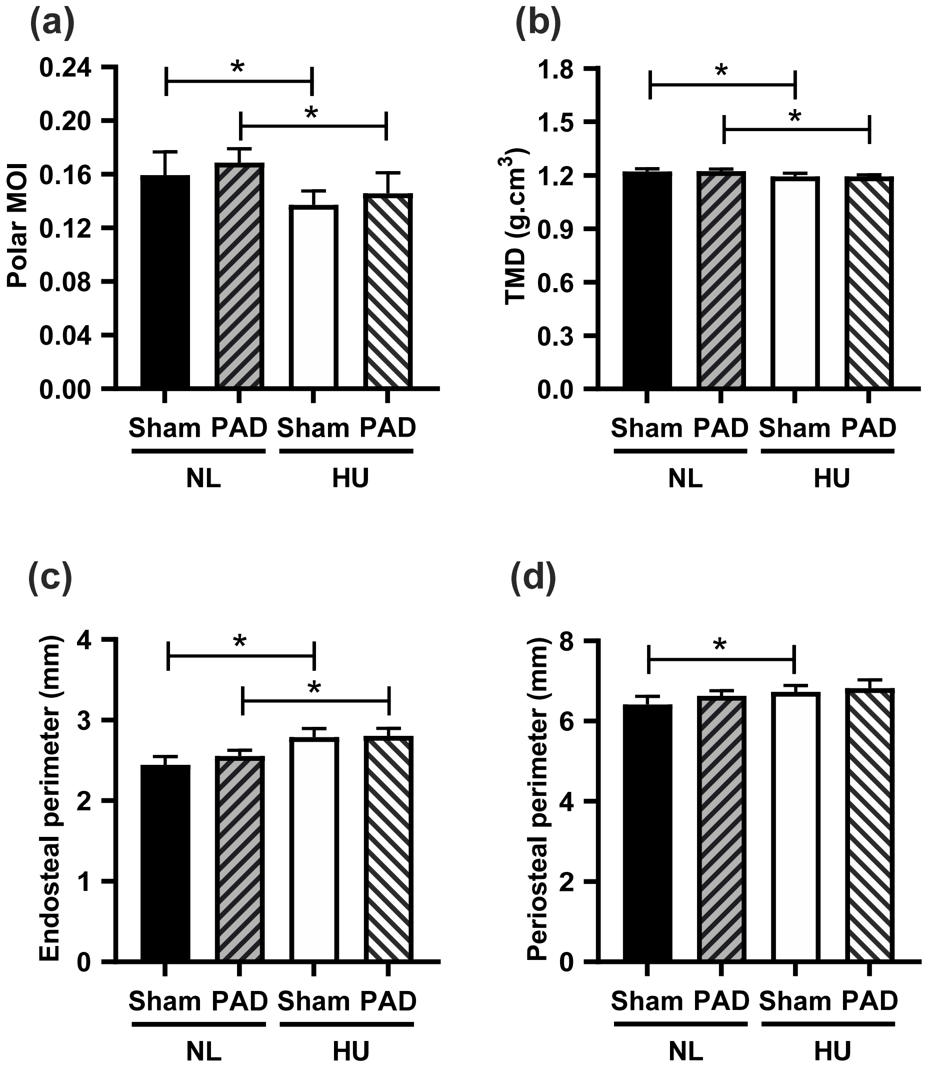

Supplementary Figure 2. Microcomputed tomography analysis of cortical bone of tibia at 30 days post-HU. (a) Polar Moment of Inertia, pMOI; (b) Tissue Mineral Density, TMD; (c) Endosteal Perimeter, Es.Pm.; and (d) Periosteal Perimeter, Ps.Pm. NL Sham (n=11), NL PAD (n=12), HU Sham (n=11), and HU PAD (n=10). (b-d). Statistically significant by one-way ANOVA and Tukey post-hoc test at $p<0.05$. (a) statistically significant by nonparametric Wilcoxon all pairs test at $p<0.05$.
