# Supplementary material for: Placenta-Expanded Stromal Cell Therapy in a Rodent Model of Simulated Weightlessness
Source: Cells. 2021 Apr 19;10(4):940. doi: 10.3390/cells10040940 (PMC8073415; doi:10.3390/cells10040940)
Supplement: Supplementary file 1 [file cells-10-00940-s001.zip › Suppl Fig 3 revised.pdf]

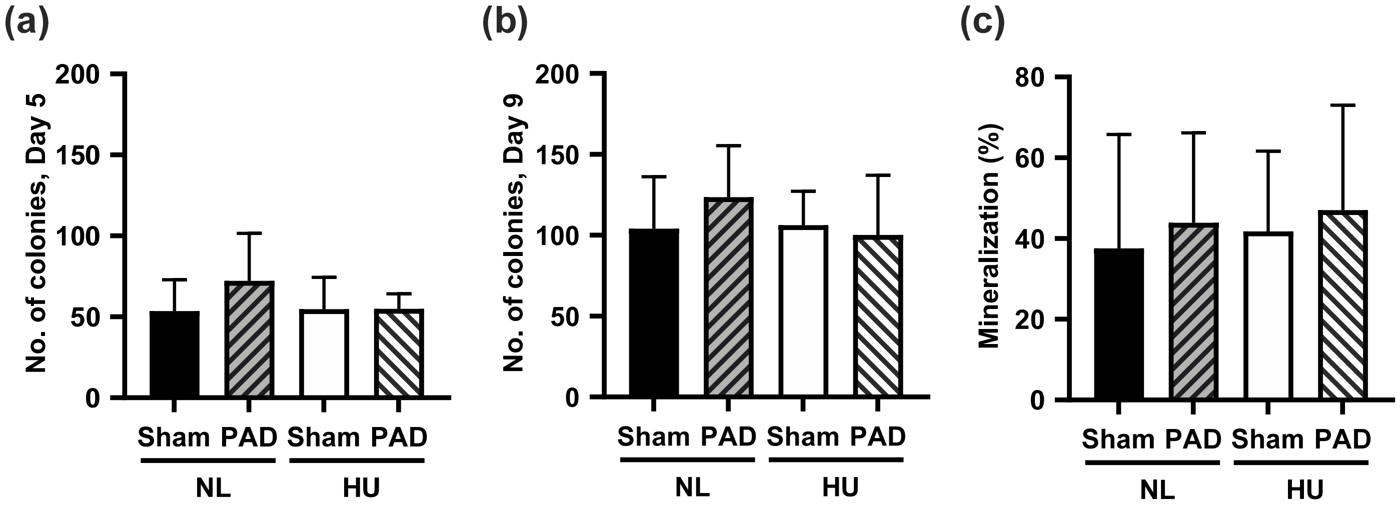

Supplementary Figure 3. *Ex vivo* osteoblastogenesis assay from bone marrow stromal cells (BMSCs) of HU and NL mice. Colonies growing on a 6 cm dish (11.55 cm^2) were scored. (a) Colony numbers at Day 5 in culture. NL Sham (n=7), NL PAD (n=6), HU Sham (n=7), and HU PAD (n=6). Statistical analysis by non-parametric Wilcoxon all pairs test at $p<0.05$. (b) Colony numbers at Day 9 in culture. NL Sham (n=7), NL PAD (n=6), HU Sham (n=7), and HU PAD (n=6). Statistical analysis by one-way ANOVA at $p<0.05$. (c) Percent mineralization following 22 days of culture in osteogenic media. Statistical analysis by nonparametric Wilcoxon all pairs test at $p<0.05$.
