# Supplementary material for: Placenta-Expanded Stromal Cell Therapy in a Rodent Model of Simulated Weightlessness
Source: Cells. 2021 Apr 19;10(4):940. doi: 10.3390/cells10040940 (PMC8073415; doi:10.3390/cells10040940)
Supplement: Supplementary file 1 [file cells-10-00940-s001.zip › Suppl Fig 4 revised.pdf]

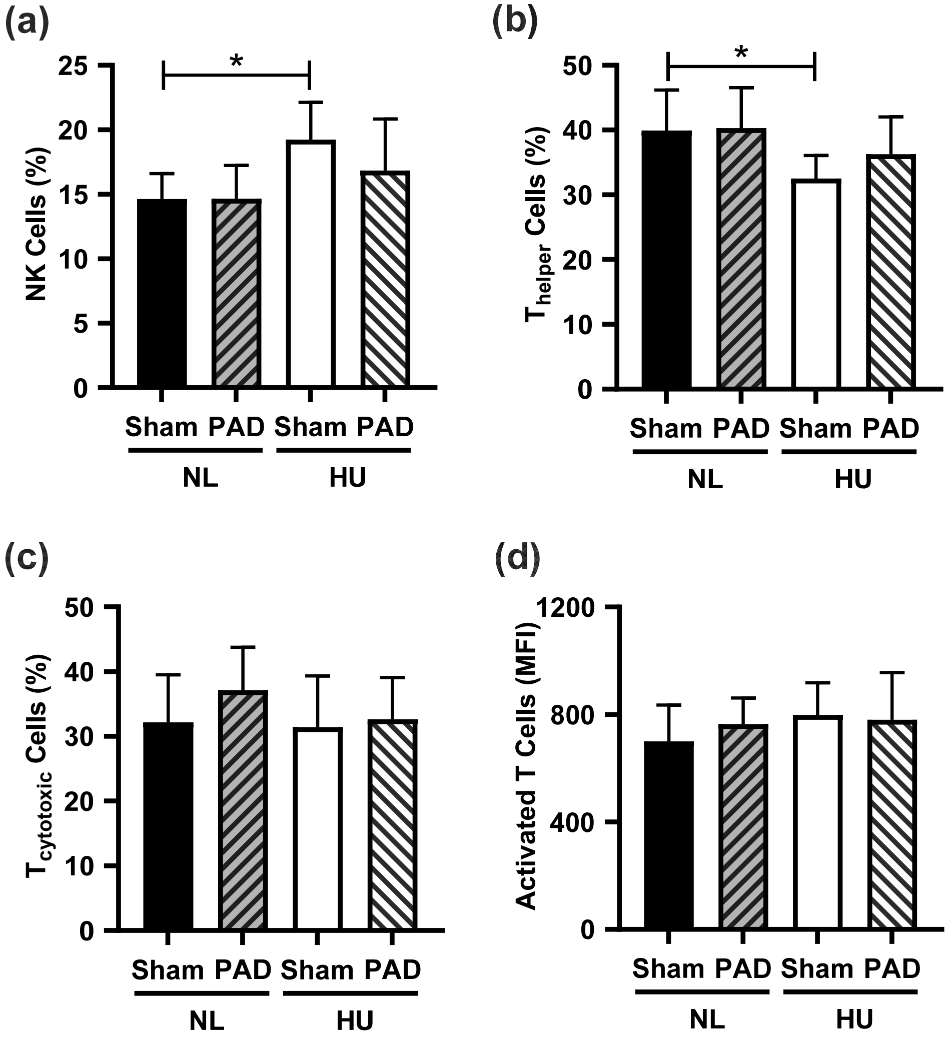

Supplementary Figure 4. Results from flow cytometry of whole blood collected from mice at day 30 post-HU. (a) Natural killer cells within leukocytes population percentages (%, NKp46+CD11b+/CD45+); (b) T helper lymphocytes within leukocyte population percentage (%, CD4+CD8-/CD3+/CD45+); (c) T cytotoxic lymphocytes within leukocyte population percentage (%, CD8+CD4-/CD3+/CD45+). NL Sham (n=12), NL PAD (n=12), HU Sham (n=11), and HU PAD (n=9); Statistical analysis by one-way ANOVA at $p<0.05$. (d) Median fluorescence intensity of CD69 (activated T cells) within total lymphocytes population percentage (MFI, CD69+/CD3+/CD45+).
