# Supplementary material for: Placenta-Expanded Stromal Cell Therapy in a Rodent Model of Simulated Weightlessness
Source: Cells. 2021 Apr 19;10(4):940. doi: 10.3390/cells10040940 (PMC8073415; doi:10.3390/cells10040940)
Supplement: Supplementary file 1 [file cells-10-00940-s001.zip › Suppl Table 2 Summary of cytokine results Final corrected.pdf]

**Supplementary Table 2.** Summary of statistical analysis of hippocampal and plasma cytokines.

A. Summary of statistical analysis of hipocampal cytokines

| | |
|---|---|
| Cytokines altered by HU (no mitigation by PLX-PAD) | EPO |
| Cytokines altered by HU and mitigated by PLX-PAD | IL-2, IL-6, IL-7, IL-13, MIG, MCSF, IL-5, MCP-1 |

B. Summary of statistical analysis of plasma cytokines

| | |
|---|---|
| Cytokines altered by HU (no mitigation by PLX-PAD) | Timp1 |
| Cytokines altered by HU and mitigated by PLX-PAD | IL-6 (high variance) |
