# Supplementary material for: Placenta-Expanded Stromal Cell Therapy in a Rodent Model of Simulated Weightlessness
Source: Cells. 2021 Apr 19;10(4):940. doi: 10.3390/cells10040940 (PMC8073415; doi:10.3390/cells10040940)
Supplement: Supplementary file 1 [file cells-10-00940-s001.zip › Supplementary Methods Final v2.pdf]

## Supplementary Methods

***Ex vivo osteoblastogenesis.*** As previously described [1], bone marrow cells were flushed out of femurs. Marrow cells were then centrifuged at 1000xg for 5 minutes. Cells were plated in T12.5 flasks at a final density of $3x10^6$ cells/$cm^2$ in osteogenic media composed of α-MEM, 15% heat deactivated fetal bovine serum, 1X Antibiotic-Antimycotic (penicillin (100 U/ml), streptomycin 100ug/ml of, Amphotericin (0.25 µg/ml), Gibco Thermofisher), 0.01M β-glycerophosphate (Sigma-Millipore), and 0.05 mM ascorbic acid (Sigma-Millipore). Media were changed every 2-3 days. On day 7 and on day 9, all of the live colonies consisting of at least 50 cells were counted using an Olympus inverted microscope at 4X magnification, with a printed grid positioned beneath the flask. Results shown in Supplementary Figure 3 are the total number of colonies counted in each T12.5 flask.

**Alizarin red staining.** At day 22 post-plating after abundant mineralized nodules were evident, osteogenic cultures were washed with PBS and fixed in 4% paraformaldehyde (FD Technologies) at room temperature for an hour. Following incubation, cells were washed in distilled water and stained with 2% Alizarin Red (Millipore-Sigma) at room temperature for 15 minutes. Excess staining solution was aspirated. Flasks were washed with Phosphate Buffered Saline Solution (PBS) to remove any residual stain and allowed to air dry overnight. Once completely dry, flasks were scanned with EPSON Perfection (1640SU) and imaged using Image Capture (version 7.0). Images were scanned and then processed for percent area mineralization using ImageJ (version 2.0.0-rc-69/1.52p) and values were averaged between technical duplicates.

## Reference

1. Schreurs, A. S., Shirazi-Fard, Y., Shahnazari, M., Alwood, J. S., Truong, T. A., Tahimic, C. G., Limoli, C. L., Turner, N. D., Halloran, B., & Globus, R. K. (2016). Dried plum diet protects from bone loss caused by ionizing radiation. *Sci Rep*, *6*, 21343. https://doi.org/10.1038/srep21343
